# Neural variability across the transition to motherhood: Enhanced moment‐to‐moment neural variability during mentalizing in first‐time mothers

**DOI:** 10.1111/jne.70191

**Published:** 2026-04-30

**Authors:** Sara Halmans, Milou Straathof, Sophie van ’t Hof, Damiaan Denys, Eveline A. Crone, Kristoffer N. T. Månsson, Elseline Hoekzema

**Affiliations:** ^1^ Pregnancy Brain Lab, Department of Psychiatry, Amsterdam Neuroscience, Amsterdam Reproduction and Development Amsterdam University Medical Center (AUMC) Amsterdam The Netherlands; ^2^ Department of Psychiatry AUMC, University of Amsterdam Amsterdam The Netherlands; ^3^ Brain and Development Research Centre, Leiden Institute for Brain and Cognition Leiden University Leiden The Netherlands; ^4^ Erasmus School of Social and Behavioral Sciences Erasmus University Rotterdam Rotterdam The Netherlands; ^5^ Centre for Psychiatry Research, Department of Clinical Neuroscience Karolinska Institutet Stockholm Sweden

**Keywords:** mentalizing, neural variability, parity, pregnancy, social cognition

## Abstract

Pregnancy is known to induce profound structural adaptations in the female brain, especially in regions involved in social cognition. This pre‐conception cohort fMRI study examined changes in neural signal variability and functional connectivity during mentalizing tasks among 110 women (M_age_ = 30.5 years, SD = 3.5, range = 25–41), including 40 first‐time mothers, 30 s‐time mothers, and 40 nulliparous control women. Participants completed a mentalizing task before and after pregnancy, and, for a subset, 1 year postpartum. First‐time mothers exhibited increased neural variability in response to child‐related stimuli in the early postpartum period relative to their pre‐conception baseline, whereas control women and second‐time mothers showed decreases consistent with typical age‐related changes. In controls, decreased neural variability correlated with declines in mentalizing task performance, suggesting that neural variability supports flexible and effective cognitive processing. Effects were stimulus‐ and parity‐specific, with first‐time mothers showing selective increases to child‐related cues and second‐time mothers showing distinct changes in adult‐related processing. These findings suggest that pregnancy, particularly in first‐time mothers, selectively preserves or enhances neural flexibility for processing infant social cues and that neural variability is a key marker of these adaptations.

## 1 INTRODUCTION

Pregnancy marks a profound neuroendocrine transition in women, characterized by significant structural and functional adaptations in the brain.^1^, ^2^, ^3^, ^4^ Notably, extensive reductions in gray matter volumes have been observed, particularly in the anterior and posterior cortical midline and specific regions of the bilateral lateral prefrontal and temporal cortex.^1^, ^2^ These regions closely overlap with the neural network supporting mentalizing, the capacity to infer thoughts, intentions, and emotions of others. This capacity has also been termed theory of mind (ToM) or cognitive empathy and is encompassed within the broader concept of social cognition; but following recent consensus work on the employed terminology in the field,^5^ we use the term “mentalizing” throughout this study. Mentalizing is fundamental for effective maternal behavior.^6^, ^7^, ^8^ Mothers need to frequently interpret subtle cues from their infants, relying on mentalizing to respond appropriately to their needs. Observational evidence suggests that mothers who comment appropriately on their infants' psychological states and object‐directed activity during the first months of life foster more secure attachment relationships.^9^, ^10^, ^11^ Moreover, social cognition capacity has been linked to maternal sensitivity and lower levels of depressive interactional behaviors.^6^, ^7^

Neuroimaging findings further highlight the connection between changes in social‐cognitive brain networks and maternal adaptation. Analysis of functional magnetic resonance imaging (fMRI) data demonstrated a strong overlap between the brain regions undergoing pregnancy‐related gray matter volume reductions and those showing neural activation to pictures of their own infants after childbirth.^1^ Other work has shown that mothers, compared to nulliparous women, exhibit greater activation in regions typically associated with ToM and empathic processing when viewing infant emotional cues,^12^ and more broadly in areas supporting social understanding regardless of whether stimuli are adult or child‐related.^13^ Notably, these studies examine activation patterns in mentalizing‐associated brain regions rather than employing tasks specifically targeting mentalizing. More broadly, parental brain research has predominantly probed social cognition through child‐specific cues, whereas social neuroscience relies on standardized measures such as the Reading the Mind in the Eyes Test or false‐belief paradigms, which might be limiting cross‐field comparability. By applying such a standardized measure to a parental sample, the present study enables findings to be interpreted within and translated across the wider mentalizing literature. Together, these findings suggest that brain networks typically implicated in mentalizing are dynamically recruited during early motherhood in ways that may support sensitive and adaptive caregiving.

Although the neural changes associated with pregnancy have been increasingly documented, research specifically examining mentalizing during the transition to motherhood remains limited. Recent behavioral studies suggest that while self‐reported mentalizing abilities may remain unchanged across pregnancy, improved performance has been observed on one task assessing a construct related to mentalizing.^14^ These findings point to adaptive modifications in social‐cognitive brain networks, potentially supporting the complex demands of maternal caregiving.

In parallel, while functional neuroimaging research has traditionally focused on mean signal changes, such as the heightened mentalizing‐related activation observed in first‐time mothers, neural variability has emerged as a complementary and sensitive marker of neural function and adaptability. Across the lifespan, neural variability follows an inverted U‐shaped trajectory, peaking in early adulthood (22–31 years) and declining with age.^15^, ^16^, ^17^ This peak in neural variability during early adulthood is particularly relevant for understanding neural function in first‐time mothers, who typically fall within this age range. Greater variability has been linked to enhanced cognitive flexibility and faster behavioral responses, underscoring its relevance for efficient information processing.^15^, ^18^, ^19^ Intriguingly, recent evidence indicates that while neural variability during resting‐state fMRI remains stable across pregnancy, it declines in nulliparous controls over a comparable time interval,^20^ suggesting that pregnancy may help preserve neuroplasticity with aging. Task‐based neural variability has demonstrated excellent test–retest reliability over extended periods (e.g., 11 weeks), indicating its stability as a neural marker.^21^ Despite these advances, the interplay between neural variability and mentalizing, particularly in the context of pregnancy, remains unexplored. Investigating this relationship could yield critical insights into the neural mechanisms underlying mentalizing and associated maternal adaptation during the peripartum period.

In this study, we aimed to investigate whether pregnancy is associated with changes in neural signal variability during mentalizing‐related cognitive processing. We analyzed task‐based fMRI data collected during mentalizing paradigms from a longitudinal sample of 110 women drawn from a prospective pre‐conception cohort,^2^, ^22^ including first‐time mothers (*n* = 40), second‐time mothers (*n* = 30), and nulliparous controls (*n* = 40). Women in the first‐ and second‐time pregnancy groups were scanned before conception, at an early and late postpartum time point, and control women were followed over a similar time interval. We hypothesized that, consistent with the broader adaptive reorganization of social‐cognitive brain networks during the transition to motherhood, pregnancy would be associated with increases in task‐based neural variability in mentalizing‐related regions. Given evidence that neural variability during resting‐state fMRI is preserved in pregnant women but declines in nulliparous women,^20^ we further hypothesized that nulliparous controls would show a relative decline in task‐based neural variability over the same period. Finally, given that second pregnancies represent a repeated neuroendocrine transition in women who have already undergone maternal brain adaptations, we explored whether the pattern of variability changes would differ between first‐ and second‐time mothers.

## 2 METHODS

### 2.1 Design and participants

Data were drawn from a prospective pre‐conception cohort study conducted in Leiden, the Netherlands, with ethical approval from the Leiden University Medical Centre. The sample included nulliparous women (planning or not planning pregnancy within 1 year) and primiparous women planning a second pregnancy. Group assignments were based on pregnancy intentions and outcomes, resulting in three groups: control (CTR), first‐time pregnancy (PRG1), and second‐time pregnancy (PRG2). Structural and functional MRI scans were performed before conception (Pre) and in the early postpartum period, approximately 3 months postpartum (Post); a subset of the PRG1 and PRG2 groups also had a follow‐up MRI scan 1 year postpartum (Post +1y).

Demographic information of the sample can be found in Table 1. Age differed significantly across groups, with the PRG2 group being older than both the CTR and PRG1 groups; no significant age difference was found between CTR and PRG1 (PRG1: 29.74 ± 3.58 years; PRG2: 32.5 ± 2.25 years; CTR: 29.7 ± 3.61 years). No significant between‐group differences were observed for education level, gestation length, or the Pre‐to‐Post or Parturition‐to‐Post time interval. For further details on participant recruitment and inclusion criteria, please refer to the paper of Halmans et al.^20^

**TABLE 1:** Demographics.

Characteristics	PRG1	PRG2	CTR	Between Group Differences
Sample size	40	30	40	
Post +1 year	28	14	‐	
Age at pre (*M* ± SD years)	29.74 ± 3.58	32.5 ± 2.25	29.7 ± 3.61	*F*(2,106) = 7.547, *p* < 0.001* *CTR* versus *PRG1: W* = 792, *p* = 0.94 *CTR* versus *PRG2: W* = 254, *p* < 0.001* *PRG1* versus *PRG2: W* = 256, *p* < 0.001*
Education (Verhage score)	6.43 ± 0.78	6.33 ± 0.76	6.65 ± 0.53	*F*(2,107) = 1.995, *p* = 0.14
Duration Pre‐Post (*M* ± SD days)	509.78 ± 158.9	502.7 ± 125	457.9 ± 81.9	*F*(2,107) = 1.934, *p* = 0.15
Duration Parturition‐Post (*M* ± SD days)	100.63 ± 70.8	80.32 ± 27.7	‐	*W* = 611, *p* = 0.53
Duration Parturition‐Post +1y (*M* ± SD days)	402.58 ± 64.8	408.67 ± 38.7	‐	*W* = 70, *p* = 0.72
Gestation length at birth (weeks)	39.05 ± 2.4	39.48 ± 1.33	‐	*W* = 525.5, *p* = 0.80
Fetal sex				
Male	17	15	‐	
Female	22	13	‐	
Twins	1	0	‐	

*Note*: PRG1 = nulliparous women who became pregnant between the sessions, PRG2 = primiparous women who became pregnant with their second child between the sessions, CTR = nulliparous women who did not become pregnant between the sessions, *M* = mean, SD = standard deviation, *F* = test statistic from an analysis of variance, *W* = test statistic from the Wilcoxon rank sum test. The Verhage score is a measure of educational level based on the Dutch educational system, ranging from 1 (less than primary school) to 7 (academic degree); scores of 6 or 7 are classified as high educational level. Regarding fetal sex, for each group, an exact binomial test against a 50:50 distribution was non‐significant (*p* > 0.05). * *p* < 0.05.

### 2.2 MRI data acquisition

The functional MRI scans used in this study were acquired during two mentalizing tasks (see description below). T2*‐weighted whole‐brain echoplanar images (EPIs) were obtained with a 3‐Tesla Philips MRI scanner. We acquired 186 volumes (including two dummy scans to allow for equilibration of T1 saturation effects) for each mentalizing task with the following parameters: repetition time (TR) = 2.2 s; echo time (TE) = 30 ms; flip angle = 80°; field of view = 220 × 220 × 111.65 mm; voxel size = 2.75 × 2.75 × 2.75; 37 descending slices. Additionally, high‐resolution 3D T1‐weighted images were acquired in transverse orientation (TR = 9.8 ms; TE = 4.6 ms; flip angle = 8°; voxel size = 0.875 × 0.875 × 1.20 mm^3^; field of view = 178 × 224 × 168 mm).

### 2.3 Mentalizing fMRI task

To investigate the neural signal variability during mentalizing processes, the Reading the Mind in the Eyes task,^23^ including adult stimuli, and the Reading the Mind in the Child's Eyes task^24^ were used during an fMRI scan. E‐Prime 3.0 software (Psychology Software Tools, Pittsburgh, PA) was used to present greyscale pictures of adult's or children's eyes expressing an emotion. Two emotion‐describing words (e.g., “wanhopig” and “verlegen” [Dutch for “desperate” and “shy”]) were presented on the left and right below the picture and the participants were instructed to choose as fast as possible which of the two words they associate with the shown picture (see Figure S1), using a button box within the scanner. An adapted fMRI version of the task was used, that presents two words instead of four.^25^ As a control condition, the participants were asked to determine the gender of the people on the same pictures shown. It was randomized whether the participants did the adult task or the children task first and within each of these tasks blocks of the mentalizing condition and the control condition were always alternated. This led to the four conditions mentalizing adult, mentalizing child, control adult and control child. The task consisted of 30 trials per condition, separated into six blocks, and each trial was presented for 5 s, after instructions about which task had to be performed (2 s).

### 2.4 Functional MRI data preprocessing and neural variability estimation

Prior to the preprocessing steps, the MRI scans were visually checked for gross artifacts such as signal dropout, geometric distortion, and insufficient brain coverage. No scans were excluded. This resulted in a complete Pre and Post dataset for 40 participants in the CTR group and 40 participants in the PRG1 group, with 28 of the latter also completing the Post +1y session. In the PRG2 group, 30 participants had complete Pre and Post datasets, and 14 of these women also participated in the Post +1y session.

First, functional MRI data were preprocessed using fMRIPrep (version 23.1.2), FSL (version 6.0.3), SPM12 (v7771), and MATLAB (version 9.12.0, R2022a). T1‐weighted images served for registration and gray matter masking. The main preprocessing steps of the fMRI data included brain extraction, correction for head motion, spatial smoothing with a 7 mm FWHM Gaussian kernel, and bandpass filtering between 0.01 and 0.1 Hz. Second, independent component analysis (ICA) was performed for each subject and time point using MELODIC in FSL. Third, a manual denoising process following Griffanti et al.^26^ was applied to identify and remove noise components from the data. Two trained researchers independently classified each component as either “signal” or “noise”; if there was disagreement or uncertainty, a third highly trained researcher provided the final classification. Identified noise components were regressed out of the time series using the regfilt command in FSL. Fourth, functional images were then normalized to MNI152NLin6Asym standard space (resampled to 3 mm isotropic resolution). Finally, the Variability Toolbox (VarTbx) in SPM12 was used to calculate the temporal standard deviation of the BOLD signal (SD_BOLD_) voxel‐wise of the mentalizing conditions for each subject at each time point. All blocks within each condition were concatenated and SD_BOLD_ was computed across the concatenated time series for each subject.

### 2.5 Statistical analyses

Statistical analyses were conducted using SPM12 (v7771; https://www.fil.ion.ucl.ac.uk/spm), implemented in MATLAB (version 9.12.0, R2022a), and R Statistical Software in RStudio (v4.2.1). Group‐by‐time interaction effects in neural variability were modeled in SPM12 using a full factorial design. First, the Pre and Post time points of the PRG1 group were compared to the CTR group, then to the PRG2 group and lastly the CTR group to the PRG2 group. Interaction effects were assessed with F‐contrasts and evaluated at peak‐level at a family‐wise error (FWE) corrected threshold of *p* < 0.05 and an extent threshold of 10 voxels. For within‐group comparisons, paired *t*‐tests were applied for all time point combinations (Pre vs. Post, Post vs. Post +1y and Pre vs. Post +1y). Here, two T‐contrasts were applied to investigate potential changes in both directions. For the post‐hoc tests, no comparisons survived the FWE‐correction, which is why we applied an uncorrected *p*‐value of <0.001 for exploratory purposes. To assess baseline differences, two‐sample *t*‐tests were applied to compare neural variability at the Pre session between the groups, with two T‐contrasts assessing differences in both directions. All reported coordinates are in MNI standard space, and anatomical descriptions are provided according to the Automated Anatomical Labeling atlas (AAL3 implementation in SPM). All between‐group analyses involving the PRG2 group were conducted with age as covariate to account for group differences in age. For completeness, models without including age as a covariate are also reported in the Supporting Information. The interval between birth and the Post session did not differ between the PRG groups and was therefore not included as a covariate in primary analyses; robustness checks including this interval confirmed the main findings. Additionally, fetal sex, number of children and gestation length were examined as potential covariates for neural variability at the Post session. None of these variables showed a significant association and were therefore not included in the primary analyses.

Statistical testing in R was preceded by assessment of distributional assumptions. Normality was checked using the Shapiro–Wilk test, and homogeneity of variances was examined with Levene's test. Parametric analyses were performed when assumptions were met, including independent and paired *t*‐tests as well as Pearson correlations (reported as *r*). Non‐parametric alternatives were used when assumptions were violated, involving Wilcoxon rank‐sum tests for group comparisons and Spearman correlations (reported as *rho*). Outliers, defined as values exceeding ±2 standard deviations from the mean, were excluded prior to conducting the analyses.

During the mentalizing tasks, behavioral performance was measured based on the accuracy of identifying the correct expression. Mixed‐effects models, paired and two‐sample *t*‐tests, and Wilcoxon rank‐sum tests were used to assess differences in mentalizing performance between groups, time points, and conditions. Correlation analyses for the mentalizing child condition were performed to examine how the different changes of neural variability over time within the groups related to changes in mentalizing performance. Therefore, the change in accuracy over time (Post minus Pre) was calculated and correlated with the changes in the left calcarine gyrus cluster resulting from the significant interaction between the CTR and the PRG1 group. Performance outliers (defined as above, ± 2 standard deviations from the mean) were removed, resulting in *n* = 38 for the PRG1 group and *n* = 37 for the CTR group.

To explore if task‐based neural variability showed a similar association with aging processes as seen in our resting‐state data, whole‐brain mean neural variability values were extracted using a gray matter mask in FSL and then correlated with age at baseline.

## 3 RESULTS

### 3.1 Mentalizing child

#### 3.1.1 Neural variability changes in first‐time mothers

**FIGURE 1:**
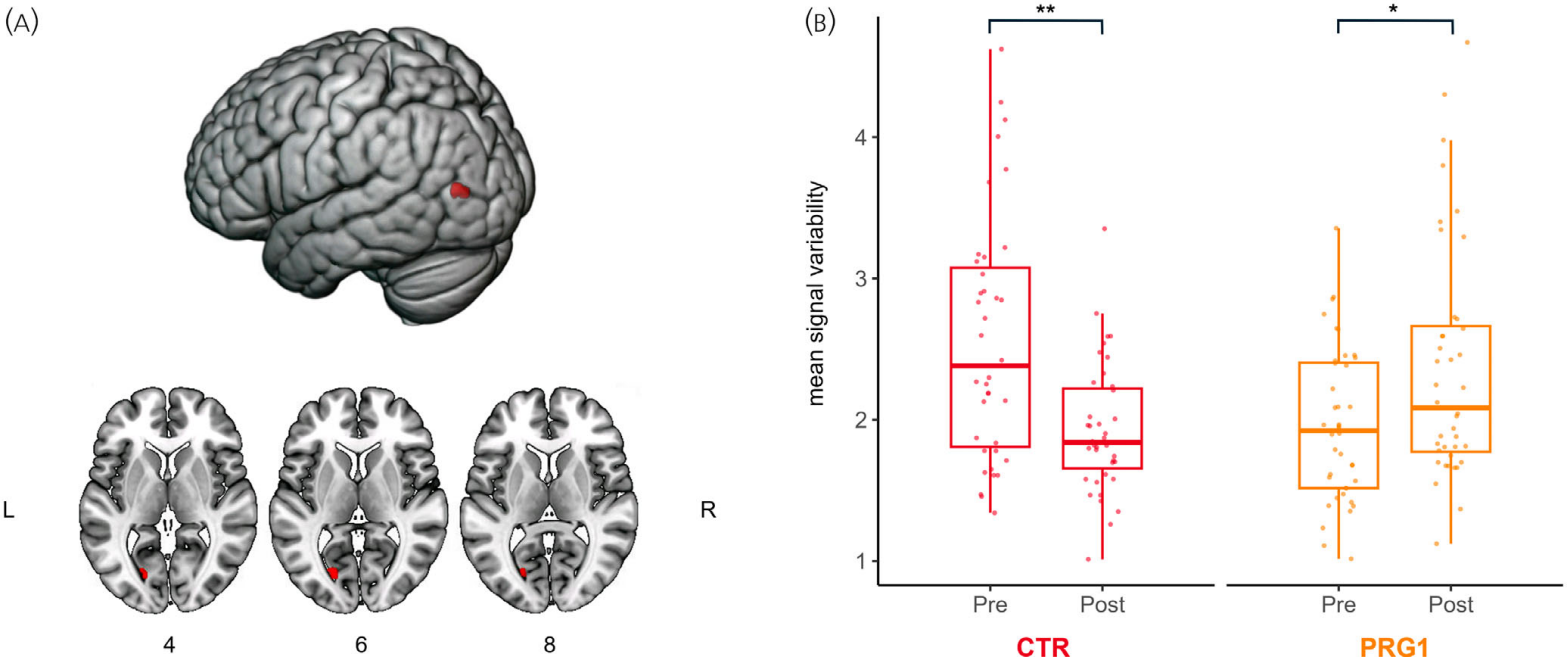
Neural variability changes over time in the group‐by‐time interaction cluster in the left calcarine gyrus during the mentalizing child condition. Visualization of the cluster location from the interaction model for CTR versus PRG1 and the group distribution per time point. CTR = control group (*n* = 40), PRG1 = first‐time pregnancy group (*n* = 40), Pre = before conception, Post = early postpartum. (A) shows the location of the interaction cluster. (B) shows the mean neural variability per time point for each group in this cluster. Boxplots show the median and interquartile range (IQR); whiskers extend to 1.5× IQR. Individual data points (jittered) are overlaid for visualization and include all values. **p < 0.001, *p < 0.05.

To investigate whether becoming a mother is associated with changes in neural variability, we compared the changes in variability from Pre to Post between the CTR and the PRG1 group. When conducting a voxel‐wise whole‐brain analysis of the mentalizing child data checking for possible interactions between group and time, we found a significant interaction in the left calcarine gyrus (MNI *xyz* = −21, −72, 6; *k* = 324 mm^3^, *F* = 34.6, *p*_FWE_ = 0.001). This interaction effect shows a decrease of neural variability in the CTR group and an increase in the PRG1 group. Extracted mean neural variability of this cluster for each group and time point, along with the cluster locations, is presented in Figure 1.

**FIGURE 2:**
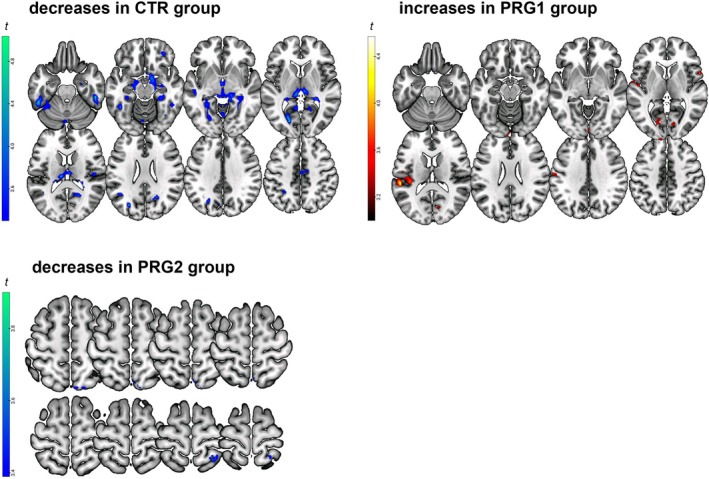
Within‐group comparisons of whole‐brain neural variability over time for the control and pregnancy groups during the mentalizing child condition. Paired *t*‐tests within groups. The left panels showing decreases in the CTR and PRG2 groups are reflecting the contrast Pre > Post, the upper right panel showing increases in the PRG1 group is reflecting the contrast Post > Pre. CTR = control group (*n* = 40), PRG1 = first‐time pregnancy group (*n* = 40), PRG2 = second‐time pregnancy group (*n* = 30), Pre = before conception, Post = early postpartum.

Post‐hoc whole‐brain within‐group comparisons were conducted per group. For the CTR group, a widespread pattern of decreases in mentalizing child neural variability from Pre to Post was observed in different regions across the brain, including the bilateral precuneus and inferior temporal gyri, the fusiform gyri, and the right hippocampus. The PRG1 group showed the opposite, with peak‐level increases in neural variability located in the calcarine gyri, the left superior temporal gyri, the right inferior frontal operculum, and the left rolandic operculum. The identified clusters for these groups are displayed in Figure 2. The opposite contrasts (increase in the CTR group, decrease in the PRG1 group) did not yield any significant peak‐level activations. Detailed information for all peak activations of both groups is provided in Tables S1 and S2.

To detect baseline differences, we compared the two groups at the Pre session with each other, rendering no significant differences.

#### 3.1.2 Neural variability in second‐time mothers

To assess whether neural variability changes similarly across a second pregnancy, we compared the PRG2 group with the CTR group and the PRG1 group. Given that the PRG2 group was significantly older than the other two groups, age‐corrected models were used as the primary analyses. Voxel‐wise whole‐brain interaction analyses revealed no significant interactions for either CTR versus PRG2 or PRG1 versus PRG2.

For completeness, within‐group changes in PRG2 were also assessed, showing four peak‐level decreases in neural variability in the right precuneus and the right superior parietal gyrus. The identified clusters for these groups are displayed in Figure 2. No increase in neural variability was observed. Detailed information about the peak activations can be found in Table S3.

#### 3.1.3 Neural variability changes in the late postpartum period

Additionally, we analyzed the changes between the pre‐conception and early postpartum time points and the late postpartum time point (Post +1y) for the two PRG groups. For the PRG1 group, we found an increase in neural variability from Pre to Post +1y, with four peak‐level activations in the left temporal and supramarginal gyri. When assessing mean neural variability within the PRG1 interaction cluster, the difference between Pre and Post +1y was not statistically significant (*V* = 134, *p* = 0.07) nor between Post and Post +1y (*V* = 279, *p* = 0.19), suggesting a slight but nonsignificant reduction during the postpartum period that does not reach the baseline level. The PRG2 group showed a small increase in the right insula. For both groups, there were no peak‐level activations for the opposite contrasts as well as for the comparison between Post and Post +1y. Full details of the peak‐level activations for these comparisons are available in Tables S4 and S5.

### 3.2 Mentalizing adult

#### 3.2.1 Neural variability changes in first‐time mothers

Voxel‐wise whole‐brain interaction analyses of neural variability during the mentalizing task with adult stimuli revealed no significant interaction effect between the CTR and the PRG1 group.

#### 3.2.2 Neural variability changes in second‐time mothers

With age included as covariate, interaction analyses revealed no significant interactions for either CTR versus PRG2 or PRG1 versus PRG2. Models without correcting for age are reported in Table S6 and Figure S2.

Within‐group post‐hoc comparisons in the CTR group showed 10 significant peak‐level activations reflecting a decrease of neural variability from Pre to Post in the left middle temporal gyrus, the fusiform gyri, and the right hippocampus. For the PRG1 group, we observed decreases of neural variability from Pre to Post in the left superior parietal and precentral gyrus, the right precuneus, and the left hippocampus. The opposite contrasts did not reveal any significant peak‐level activations. In the PRG2 group, we observed opposite effects than in the other two groups, with four significant peak‐level activations located in the right superior frontal gyrus and the right lingual gyrus reflecting an increase of neural variability over time. There was no significant decrease over time in the PRG2 group. We did not find significant baseline differences between the groups. Detailed information about the peak activations is provided in Tables S7–S9.

#### 3.2.3 Neural variability changes in the late postpartum period

Comparisons with the Post +1y time point in the PRG groups revealed significant decreases of neural variability between Pre and Post +1y in the PRG1 group, which were located in the left precuneus, the left precentral and parahippocampal gyrus, and the left amygdala. There was no increase in neural variability and no difference between the Post and Post +1y time points. The PRG2 group revealed significant peak‐level activations showing an increase in neural variability for both Pre versus Post +1y and Post versus Post +1y, located in the right hippocampus and the left insula. When assessing mean neural variability within the PRG2 interaction cluster, we found a significant increase between Pre and Post +1y (*t*(13) = −2.79, *p* = 0.02) and no significant difference between Post and Post +1y (*t*(13) = −0.21, *p* = 0.84), suggesting a stable level of neural variability during the postpartum period. Full details of the peak‐level activations for these within‐group comparisons are available in Tables S10–S12.

### 3.3 Mentalizing performance and relationship with social cognition related neural variability

We conducted mixed‐effects models to examine changes in mentalizing performance across time points and between groups. For the mentalizing adult condition, the analysis revealed a significant main effect of the time points (*F*(1,106) = 4.33, *p* = 0.04), indicating a significant difference between the pre‐conception and early postpartum time points. Neither the main effect of the group nor the group‐by‐time interaction reached statistical significance, suggesting that the three groups did not differ significantly in their overall performance or pattern of change over time. For the mentalizing child condition, the mixed‐effects model revealed no significant effects (see Table 2 for detailed results).

**TABLE 2:** Interaction analysis of mentalizing performance of all three groups.

	Mentalizing adult		Mentalizing child	
	*F*	*p*	*F*	*p*
Intercept	5630.9	<0.001*	4037.8	<0.001*
Group	0.92	0.40	1.62	0.20
Time point	4.33	0.04*	1.55	0.22
Group × Time point	1.86	0.16	1.60	0.21

*Note*: Results of the mixed‐effects interaction model of the mean neural variability per group per time point for both task conditions. Groups included: CTR = nulliparous control group (*n* = 40), PRG1 = first‐time pregnancy group (*n* = 40), PRG2 = second‐time pregnancy group (*n* = 30); Time points included: Pre = before conception, Post = early postpartum. Degrees of freedom per model = 106. *p < 0.05.

To further characterize the pattern of change in mentalizing over time within each group, we conducted exploratory within‐group comparisons for the mentalizing adult condition. These analyses revealed that the CTR group showed a significant increase in accuracy between Pre and Post time points (*t*(39) = −2.16, *p* = 0.037), while both PRG groups did not show significant changes. Comparisons between mentalizing conditions revealed significantly higher accuracy in the mentalizing adult condition compared to the mentalizing child condition across all groups and time points. Detailed results of all pairwise comparisons are presented in Supplementary Tables S13–S15.

**FIGURE 3:**
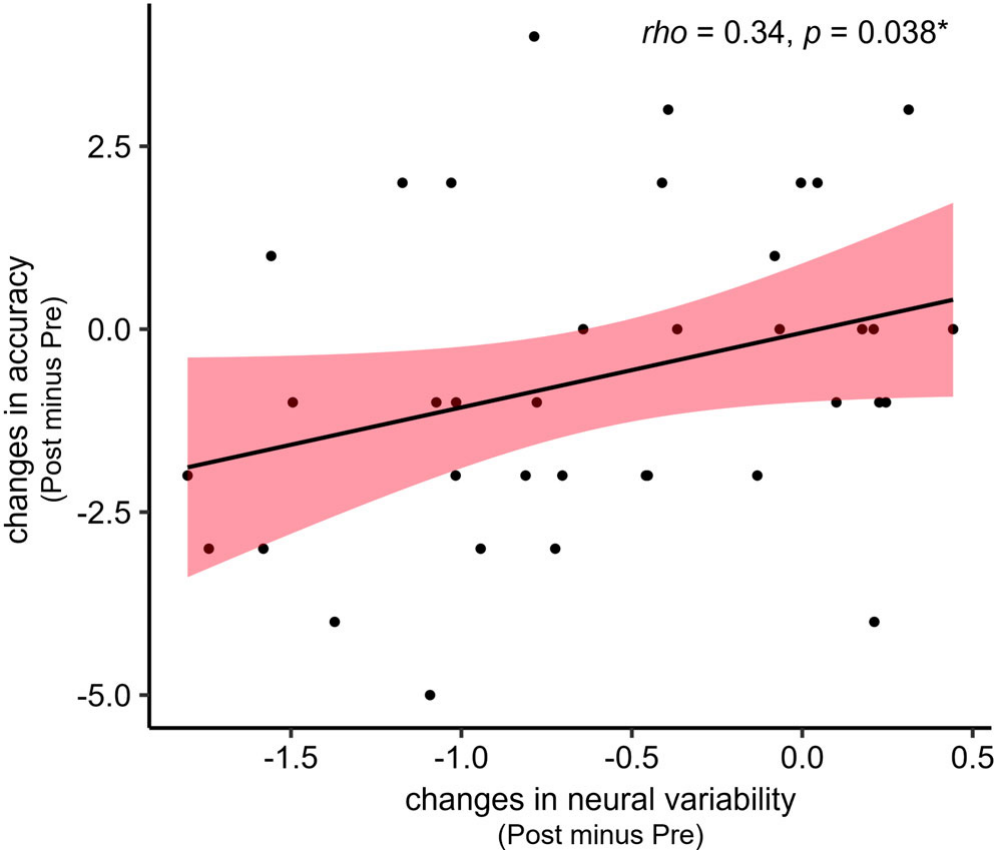
Correlation of changes in mentalizing child neural variability with changes in mentalizing performance in the control group. Correlation of neural variability changes in the significant interaction cluster (CTR vs. PRG1 interaction) with the changes in accuracy in the CTR group. CTR = control group, Pre = before conception, Post = early postpartum. Spearman correlations, *n* = 37, *p < 0.05.

To assess whether the differential changes in neural variability between the CTR and PRG1 groups were associated with changes in mentalizing performance, we focused on the cluster in the left calcarine gyrus, as this region emerged as the significant group*time interaction cluster in the neural variability analysis of the mentalizing child task. We therefore correlated mean neural variability changes in this cluster with changes in behavioral accuracy on the same task. We found no significant correlation in the PRG1 group (*r* = −0.15, *p* = 0.37). The CTR group showed a significant positive correlation (*rho* = 0.34, *p* = 0.038), suggesting that a decrease of neural variability in this cluster is associated with a decrease in performance accuracy (see Figure 3).

### 3.4 Relationship of neural variability with age

We performed correlation analyses between age and mean whole‐brain neural variability during the mentalizing conditions at the Pre session. As expected, in the whole sample (CTR, PRG1 and PRG2 combined), higher age was associated with lower neural variability at Pre for both mentalizing adult (*rho* = −0.44, *p* < 0.001) and mentalizing child conditions (*rho* = −0.23, *p* = 0.01).

## 4 DISCUSSION

In this study, we investigated the effects of pregnancy on moment‐to‐moment neural variability during mentalizing‐related cognitive processing. The first part of the analysis focused on cognitive processing of child‐related stimuli. Here we showed that neural variability decreased over time in the control group and the group of second‐time mothers, while it increased in the group of first‐time mothers. Additionally, we showed that the decrease in neural variability during mentalizing was associated with a decrease in mentalizing performance in the control group only. When focusing on cognitive processing during mentalizing with adult‐related stimuli, we found that neural variability decreased over time in the control group and the group of first‐time mothers, while it increased in the group of second‐time mothers.

One of our key findings was the different development of neural variability over time in the control group and first‐time mothers, when presented with child‐related stimuli. First‐time mothers showed an increase in neural variability at the early postpartum stage compared to the pre‐pregnancy time point. Several reports have shown that higher neural variability is associated with higher task performance and especially higher neural flexibility.^15^, ^16^, ^18^, ^27^, ^28^ This increase in neural variability during pregnancy may indicate adaptive reorganization processes that support the developing maternal brain. Notably, despite the increase in neural variability, task accuracy did not change significantly between the pre‐pregnancy and early postpartum time points in first‐time mothers, which could potentially be related to a ceiling effect. Given that the task shows limited test–retest reliability,^29^ this stability in performance may partly reflect measurement constraints, while also suggesting that the observed variability increase serves a compensatory or preparatory function to maintain behavioral efficiency amid substantial physiological and psychological adaptations. Furthermore, this increase was specific to the child‐related condition. When presented with adult‐related stimuli, first‐time mothers, similar to controls, showed a decrease in neural variability over time. This pattern supports the possibility that the increase in variability may reflect a selective, stimulus‐dependent adaptation, possibly related to the heightened salience and relevance of infant cues during early motherhood.

In contrast, the nulliparous control group exhibited a decline in neural variability over time, which may reflect several contributing factors. Age‐related declines in neural variability are well documented across adulthood,^16^, ^17^, ^30^, ^31^, ^32^ and consistent with this, we found that higher age was associated with lower neural variability at baseline across the whole sample. Additionally, the positive correlation between changes in neural variability and changes in mentalizing task accuracy in the control group aligns with the notion that reductions in neural variability may relate to decreases in neural flexibility and cognitive performance, as previously reported in the context of aging.^18^ However, test–retest effects may also contribute to this pattern. Repeated exposure to the scanner environment and task can reduce physiological arousal and novelty‐driven neural activity, leading to more stable and less variable neural responses over time.^17^, ^33^, ^34^, ^35^ As such, the observed decline in the control group likely reflects a combination of age‐related and methodological factors.

The present results also align with previous research showing that new mothers compared to nulliparous women show greater activation in response to emotional infant faces and in areas associated with social understanding.^12^, ^13^ However, to our knowledge, no other studies have examined changes in neural variability across pregnancy and the postpartum period. Prior work has mainly focused on increases in task‐related activation during emotional or socially relevant processing, indicating heightened responsiveness to infant cues in early motherhood.^36^, ^37^, ^38^, ^39^, ^40^, ^41^ Interestingly, the interaction effect between the first‐time mothers and control women was most prominent in the left calcarine gyrus, which is part of the primary visual cortex and mainly associated with the initial cortical processing of visual input.^42^ The decrease in the control group is consistent with age‐related declines in neural variability, as discussed above. We can speculate that the increase in first‐time mothers possibly reflects enhanced flexibility in visual processing that allows for more adaptive perception and prioritization of infant cues, thereby supporting effective caregiving and social attunement.^43^, ^44^ Particularly, these changes were selective for first‐time mothers, as second‐time mothers showed a decrease in neural variability, similar to the nulliparous control group. This suggests that a potential adaptation in neural variability is selective for first‐time mothers, maybe explained by the new demands that primiparous women experience. This also aligns with our previous findings, showing that functional coherence in the default mode network only increases across a first and not a second pregnancy.^22^ Notably, this parity‐specific pattern was not observed for adult‐related stimuli, where no significant group differences emerged after correcting for age, suggesting that neural variability adaptations in early motherhood may be selectively driven by infant‐related cues.

This investigation represents a characterization of neural variability during social cognitive processing associated with pregnancy. Several important limitations should be noted. Neural variability measures could not be collected during pregnancy, as the ethics committee did not approve scanning at this time. This is particularly relevant as recent literature showed that neural changes may follow non‐linear trajectories, with adaptations peaking during late pregnancy.^3^, ^4^ Consequently, our focus on the pre‐ and post‐pregnancy intervals may have missed some of the most pronounced transient neural shifts. Nevertheless, studies including different time point sampling strategies, including those centered on pre‐ and post‐pregnancy comparisons, consistently report substantial neural changes across the peripartum period, reinforcing the robustness of these effects despite variations in measurement timing. Additionally, the study cohort predominantly included women with higher education, which may limit extrapolation of the results to the broader population. However, as group educational levels were similar between pregnant and control participants, this variable is unlikely to have biased the main comparisons. Furthermore, the study did not collect data on race, ethnicity or gender identity. As these variables may influence the outcomes examined here, future studies should systematically collect and account for them to improve the generalizability and inclusivity of findings in this area. Lastly, we would like to recognize that there is an ongoing debate about whether the RMET is a sufficient tool for measuring Theory of Mind in behavior, mainly because of concerns regarding item‐level consistency and correctness.^29^ However, it is unlikely that these limitations substantially interfere with the validity of our findings, given that our primary focus was on neural processing patterns during the RMET, and our longitudinal within‐subject design controls for individual differences in task interpretation. Recent neuroimaging research continues to support the neural validity of mentalizing tasks including the RMET,^45^ indicating that the task reliably engages brain regions associated with social cognitive processing. Nevertheless, future studies employing other types of social cognitive tasks would be beneficial to establish the generalizability of our findings and to capture a broader range of social cognitive processes relevant to motherhood.

This study demonstrates that first‐time pregnancy induces selective increases in neural signal variability during child‐related mentalizing processing. Reductions of neural variability in control women and second‐time mothers seem to be in accordance with typical age‐related changes in individuals of this age range in other studies, also supported by observed associations with age in our study. Within the control group, individuals who showed stronger decreases in neural variability were associated with declines in mentalizing accuracy, suggesting that neural variability may support flexible and effective social‐cognitive processing. The stimulus‐ and parity‐specific nature of these effects underscores the nuanced neural adaptations of motherhood, especially when encountering infant cues. These results significantly advance our understanding of maternal brain plasticity, demonstrating that this involves alterations in neural variability during social‐cognitive processing.

## AUTHOR CONTRIBUTIONS

**Sara Halmans:** Conceptualization; methodology; formal analysis; visualization; writing – original draft; writing – review and editing. **Milou Straathof:** Methodology; formal analysis; writing – review and editing; visualization. **Eveline A. Crone:** Conceptualization; writing – review and editing. **Sophie van ’t Hof:** Writing – review and editing. **Damiaan Denys:** Writing – review and editing. **Kristoffer N.T. M** **å** **nsson:** Methodology; writing – review and editing. **Elseline Hoekzema:** Conceptualization; methodology; visualization; writing – review and editing.

## FUNDING INFORMATION

This project was supported by an Innovational Research Incentives Scheme grant (Veni, 451‐14‐036, Elseline Hoekzema) by the Netherlands Organisation for Scientific Research (NWO), a grant of the Leiden University Fund/Elise Mathilde Fund (CWB 740s/2t‐03‐2017/EM, Elseline Hoekzema) and a NARSAD grant from the Brain and Behaviour Research Foundation, USA (grant number 25312, Elseline Hoekzema) awarded to Elseline Hoekzema. Elseline Hoekzema is currently supported by an ERC Starting Grant (948031, Elseline Hoekzema) provided by the European Research Council.

## CONFLICT OF INTEREST STATEMENT

The authors declare no conflicts of interest.

## Supporting information


**Table S1.** Peak‐level activations for the paired *t*‐test Pre vs. Post of mentalizing child in the CTR group.
**Table S2.** Peak‐level activations for the paired *t*‐test Pre vs. Post of mentalizing child in the PRG1 group.
**Table S3.** Peak‐level activations for the paired *t*‐test Pre vs. Post of mentalizing child in the PRG2 group.
**Table S4.** Peak‐level activations for the paired *t*‐test Pre vs. Post + 1y of mentalizing child in the PRG1 group.
**Table S5.** Peak‐level activations for the paired *t*‐test Pre vs. Post + 1y of mentalizing child in the PRG2 group.
**Table S6.** Peak‐level activations for the group‐by‐time interaction of CTR vs. PRG2 in mentalizing child.
**Table S7.** Peak‐level activations for the paired *t*‐test Pre vs. Post of mentalizing adult in the CTR group.
**Table S8.** Peak‐level activations for the paired *t*‐test Pre vs. Post of mentalizing adult in the PRG1 group.
**Table S9.** Peak‐level activations for the paired *t*‐test Pre vs. Post of mentalizing adult in the PRG2 group.
**Table S10.** Peak‐level activations for the paired *t*‐test Pre vs. Post + 1y of mentalizing adult in the PRG1 group.
**Table S11.** Peak‐level activations for the paired *t*‐test Pre vs. Post + 1y of mentalizing adult in the PRG2 group.
**Table S12.** Peak‐level activations for the paired *t*‐test Post vs. Post + 1y of mentalizing adult in the PRG2 group.
**Table S13.** Within‐group comparisons of the accuracy in the two mentalizing conditions.
**Table S14.** Within‐group comparisons of the accuracy between the two conditions mentalizing adult and mentalizing child.
**Table S15.** Between‐group comparisons of the mentalizing child and mentalizing adult accuracy.
**Figure S1.** Reading the Mind in the Eyes paradigm. Visualization of the task used during the fMRI. There were alternating blocks of either the emotion (mentalizing) or the gender (control) condition. Each block consisted of five different stimuli. The setup is exactly the same for the Reading the Mind in the Child's Eyes task, just the pictures are showing children's eyes.
**Figure S2.** Neural variability changes over time in the group‐by‐time interaction cluster in the right fusiform gyrus during the mentalizing adult condition. Visualization of the cluster location from the interaction model for CTR vs. PRG2 and the group distribution per time point. CTR = control group (*n* = 40), PRG2 = second‐time pregnancy group (*n* = 30), Pre = before conception, Post = early postpartum. (A) shows the location of the interaction cluster. (B) shows the mean neural variability per time point for each group. Boxplots show the median and interquartile range (IQR); whiskers extend to 1.5× IQR. Individual data points (jittered) are overlaid for visualization and include all values. **p < 0.001, *p < 0.05.

## Data Availability

The data that support the findings of this study are available from the corresponding author upon reasonable request.
