# Supplementary material for: Neural variability across the transition to motherhood: Enhanced moment‐to‐moment neural variability during mentalizing in first‐time mothers
Source: J Neuroendocrinol. 2026 Apr 30;38:e70191. doi: 10.1111/jne.70191 (PMC13132796; doi:10.1111/jne.70191)
Supplement: Supplementary file 1 — Table S1. Peak‐level activations for the paired t‐test Pre vs. Post of mentalizing child in the CTR group. Table S2. Peak‐level activations for the paired t‐test Pre vs. Post of mentalizing child in the PRG1 group. Table S3. Peak‐level activations for the paired t‐test Pre vs. Post of mentalizing child in the PRG2 group. Table S4. Peak‐level activations for the paired t‐test Pre vs. Post + 1y of mentalizing child in the PRG1 group. Table S5. Peak‐level activations for the paired t‐test Pre vs. Post + 1y of mentalizing child in the PRG2 group. Table S6. Peak‐level activations for the group‐by‐time interaction of CTR vs. PRG2 in mentalizing child. Table S7. Peak‐level activations for the paired t‐test Pre vs. Post of mentalizing adult in the CTR group. Table S8. Peak‐level activations for the paired t‐test Pre vs. Post of mentalizing adult in the PRG1 group. Table S9. Peak‐level activations for the paired t‐test Pre vs. Post of mentalizing adult in the PRG2 group. Table S10. Peak‐level activations for the paired t‐test Pre vs. Post + 1y of mentalizing adult in the PRG1 group. Table S11. Peak‐level activations for the paired t‐test Pre vs. Post + 1y of mentalizing adult in the PRG2 group. Table S12. Peak‐level activations for the paired t‐test Post vs. Post + 1y of mentalizing adult in the PRG2 group. Table S13. Within‐group comparisons of the accuracy in the two mentalizing conditions. Table S14. Within‐group comparisons of the accuracy between the two conditions mentalizing adult and mentalizing child. Table S15. Between‐group comparisons of the mentalizing child and mentalizing adult accuracy. Figure S1. Reading the Mind in the Eyes paradigm. Visualization of the task used during the fMRI. There were alternating blocks of either the emotion (mentalizing) or the gender (control) condition. Each block consisted of five different stimuli. The setup is exactly the same for the Reading the Mind in the Child's Eyes task, just the pictures are showing children's eyes. Figu [file JNE-38-e70191-s001.docx]

***Supplementary Material for***

**Neural variability across the transition to motherhood: Enhanced moment-to-moment neural variability during mentalizing in first-time mothers**

Sara Halmans,^1^ Milou Straathof,^1^ Sophie van’t Hof,^1^ Damiaan Denys,^2^ Eveline A. Crone,^3,4^ Kristoffer N.T. Månsson,^5^ Elseline Hoekzema^1^

*Corresponding authors: [s.halmans@amsterdamumc.nl](mailto:s.halmans@amsterdamumc.nl) , [e.a.hoekzema@amsterdamumc.nl](mailto:e.a.hoekzema@amsterdamumc.nl)

Supplementary Tables

**Table S1**

*Peak-level activations for the paired t-test Pre vs. Post of mentalizing child in the CTR group*

| MNI coordinates | | | Cluster size (mm^3^) |  |  |  |
| --- | --- | --- | --- | --- | --- | --- |
| x | **y** | **z** |  | **T-value** | ***p*** | **Brain region** |
| 54 | -36 | -27 | 1512 | 5.31 | <0.001 | Right inferior temporal gyrus |
| 51 | -48 | -18 |  | 3.93 | <0.001 | Right inferior temporal gyrus |
| -21 | -78 | 6 | 810 | 4.94 | <0.001 | Left calcarine gyrus |
| -36 | -57 | -21 | 3780 | 4.83 | <0.001 | Left fusiform gyrus |
| -51 | -45 | -24 |  | 4.78 | <0.001 | Left inferior temporal gyrus |
| -42 | -54 | -12 |  | 4.26 | <0.001 | Left fusiform gyrus |
| 21 | -57 | 21 | 1728 | 4.73 | <0.001 | Right precuneus |
| 15 | -54 | 9 |  | 3.85 | <0.001 | Right calcarine gyrus |
| 30 | -39 | 9 | 17415 | 4.57 | <0.001 | Right hippocampus |
| -9 | -24 | 15 |  | 4.57 | <0.001 | Left thalamus |
| 3 | -18 | 15 |  | 4.55 | <0.001 | Right thalamus |
| -27 | -69 | 33 | 891 | 4.57 | <0.001 | Left middle occipital gyrus |
| -30 | -75 | 27 |  | 4.49 | <0.001 | Left middle occipital gyrus |
| -42 | -54 | 24 | 594 | 4.42 | <0.001 | Left angular gyrus |
| -48 | -27 | -6 | 351 | 4.31 | <0.001 | Left middle temporal gyrus |
| -9 | -69 | 33 | 270 | 4.23 | <0.001 | Left precuneus |
| -18 | -12 | -18 | 999 | 4.16 | <0.001 | Left hippocampus |
| -27 | -12 | -18 |  | 3.41 | <0.001 | Left hippocampus |
| -15 | -3 | -12 |  | 3.41 | <0.001 | Left amygdala |
| 9 | 9 | 9 | 270 | 4.15 | <0.001 | Right caudate nucleus |
| 36 | -57 | -18 | 378 | 4.05 | <0.001 | Right fusiform gyrus |
| 27 | -51 | -21 |  | 3.79 | <0.001 | Right cerebellum |
| 36 | -66 | -15 |  | 3.51 | <0.001 | Right fusiform gyrus |
| 48 | -18 | 15 | 270 | 4.02 | <0.001 | Right Rolandic operculum |
| 12 | -15 | 48 | 486 | 4.02 | <0.001 | Right middle cingulate gyrus |
| 3 | -15 | 45 |  | 3.85 | <0.001 | Right middle cingulate gyrus |
| -27 | -51 | 45 | 378 | 3.99 | <0.001 | Left inferior parietal gyrus |
| 33 | 42 | -15 | 324 | 3.87 | <0.001 | Right anterior orbital gyrus |
| -3 | -60 | -9 | 999 | 3.87 | <0.001 | Left cerebellum |
| -3 | -75 | -12 | 756 | 3.77 | <0.001 | Left cerebellum |
| 0 | -75 | -27 |  | 3.77 | <0.001 | Vermis |
| 6 | -84 | -27 |  | 3.54 | <0.001 | Right cerebellum |

*Note.* Peak-level activations from the t-contrast Pre > Post of the paired t-test in the CTR group, CTR = nulliparous control group (*n* = 40), Pre = before conception, Post = early postpartum (approx. 3 months).

**Table S2**

*Peak-level activations for the paired t-test Pre vs. Post of mentalizing child in the PRG1 group*

| MNI coordinates | | | Cluster size (mm^3^) |  |  |  |
| --- | --- | --- | --- | --- | --- | --- |
| x | **y** | **z** |  | **T-value** | ***p*** | **Brain region** |
| 57 | 9 | 3 | 378 | 4.72 | <0.001 | Right inferior frontal gyrus |
| -51 | -12 | 12 | 918 | 4.65 | <0.001 | Left Rolandic operculum |
| -63 | -6 | 9 |  | 3.74 | <0.001 | Left Rolandic operculum |
| -15 | -105 | 0 | 297 | 4.53 | <0.001 | Left middle occipital gyrus |
| -9 | -105 | 6 |  | 3.88 | <0.001 | Left middle occipital gyrus |
| -54 | -33 | 15 | 945 | 4.39 | <0.001 | Left superior temporal gyrus |
| -18 | -78 | 3 | 459 | 4.37 | <0.001 | Left calcarine gyrus |
| -9 | -75 | 9 |  | 3.96 | <0.001 | Left calcarine gyrus |
| -3 | -96 | -12 | 729 | 4.33 | <0.001 | Left calcarine gyrus |
| 0 | -90 | 0 |  | 3.90 | <0.001 | Left calcarine gyrus |
| -60 | -15 | 36 | 459 | 4.13 | <0.001 | Left postcentral gyrus |
| -36 | -27 | 15 | 594 | 4.13 | <0.001 | Left Rolandic operculum |
| 3 | -51 | -48 | 351 | 4.11 | <0.001 | Right cerebellum |
| 9 | -78 | 6 | 513 | 3.93 | <0.001 | Right calcarine gyrus |
| 15 | -72 | 9 |  | 3.83 | <0.001 | Right calcarine gyrus |

*Note.* Peak-level activations from the t-contrast Pre < Post of the paired t-test in the PRG1 group, PRG1 = first-time pregnancy group (*n* = 40), Pre = before conception, Post = early postpartum (approx. 3 months).

**Table S3**

*Peak-level activations for the paired t-test Pre vs. Post of mentalizing child in the PRG2 group*

| MNI coordinates | | | Cluster size (mm^3^) |  |  |  |
| --- | --- | --- | --- | --- | --- | --- |
| x | **y** | **z** |  | **T-value** | ***p*** | **Brain region** |
| 3 | -66 | 60 | 270 | 4.70 | <0.001 | Right precuneus |
| 6 | -75 | 57 |  | 4.24 | <0.001 | Right precuneus |
| 18 | -75 | 57 |  | 4.10 | <0.001 | Right superior parietal gyrus |
| 30 | -51 | 69 | 270 | 3.95 | <0.001 | Right superior parietal gyrus |

*Note.* Peak-level activations from the t-contrast Pre > Post of the paired t-test in the PRG2 group, PRG2 = second-time pregnancy group (*n* = 30), Pre = before conception, Post = early postpartum (approx. 3 months).

**Table S4**

*Peak-level activations for the paired t-test Pre vs. Post +1y of mentalizing child in the PRG1 group*

| MNI coordinates | | | Cluster size (mm^3^) |  |  |  |
| --- | --- | --- | --- | --- | --- | --- |
| x | **y** | **z** |  | **T-value** | ***p*** | **Brain region** |
| -48 | -66 | -6 | 432 | 4.84 | <0.001 | Left inferior temporal gyrus |
| -54 | -66 | 0 |  | 3.96 | <0.001 | Left middle temporal gyrus |
| -60 | -45 | 21 | 378 | 4.54 | <0.001 | Left superior temporal gyrus |
| -63 | -45 | 33 |  | 4.32 | <0.001 | Left supramarginal gyrus |

*Note.* Peak-level activations from the t-contrast Pre < Post +1y of the paired t-test in the PRG1 group, PRG1 = first-time pregnancy group (*n* = 40), Pre = before conception, Post +1y = late postpartum (approx. 1 year).

**Table S5**

*Peak-level activations for the paired t-test Pre vs. Post + 1y of mentalizing child in the PRG2 group*

| MNI coordinates | | | Cluster size (mm^3^) |  |  |  |
| --- | --- | --- | --- | --- | --- | --- |
| x | **y** | **z** |  | **T-value** | ***p*** | **Brain region** |
| 45 | 0 | 6 | 351 | 6.63 | <0.001 | Right insula |

*Note.* Peak-level activations from the t-contrast Pre < Post + 1y of the paired t-test in the PRG2 group, PRG2 = second-time pregnancy group (*n* = 30), Pre = before conception, Post +1y = late postpartum (approx. 1 year).

**Table S6**

*Peak-level activations for the group-by-time interaction of CTR vs. PRG2 in mentalizing child*

| MNI coordinates | | | Cluster size (mm^3^) |  |  |  |
| --- | --- | --- | --- | --- | --- | --- |
| x | **y** | **z** |  | **F-value** | ***p*** | **Brain region** |
| 27 | -36 | -18 | 297 | 34.43 | <0.001 | Right fusiform gyrus |

*Note.* Peak-level activations from the F-contrast of the interaction effect between the CTR and PRG2 groups without correcting for age as covariate. CTR = nulliparous control group (*n* = 40), PRG2 = second-time pregnancy group (*n* = 30).

**Table S7**

*Peak-level activations for the paired t-test Pre vs. Post of mentalizing adult in the CTR group*

| MNI coordinates | | | Cluster size (mm^3^) |  |  |  |
| --- | --- | --- | --- | --- | --- | --- |
| x | **y** | **z** |  | **T-value** | ***p*** | **Brain region** |
| -51 | -24 | -6 | 432 | 4.67 | <0.001 | Left middle temporal gyrus |
| -36 | -54 | -21 | 1539 | 4.52 | <0.001 | Left fusiform gyrus |
| -54 | -45 | -27 |  | 4.48 | <0.001 | Left inferior temporal gyrus |
| -30 | -72 | -18 | 432 | 4.41 | <0.001 | Left fusiform gyrus |
| 27 | -33 | -18 | 459 | 4.05 | <0.001 | Right fusiform gyrus |
| 27 | -33 | 15 | 378 | 4.04 | <0.001 | Right hippocampus |
| 30 | -36 | 6 |  | 3.55 | <0.001 | Right hippocampus |
| 30 | -36 | -3 |  | 3.53 | <0.001 | Right hippocampus |
| 36 | -42 | -24 | 270 | 4.01 | <0.001 | Right fusiform gyrus |
| -3 | -75 | -24 | 270 | 3.85 | <0.001 | Left cerebellum |

*Note.* Peak-level activations from the t-contrast Pre > Post of the paired t-test in the CTR group, CTR = nulliparous control group (*n* = 40), Pre = before conception, Post = early postpartum (approx. 3 months).

**Table S8**

*Peak-level activations for the paired t-test Pre vs. Post of mentalizing adult in the PRG1 group*

| MNI coordinates | | | Cluster size (mm^3^) |  |  |  |
| --- | --- | --- | --- | --- | --- | --- |
| x | **y** | **z** |  | **T-value** | ***p*** | **Brain region** |
| -24 | -75 | 42 | 891 | 4.89 | <0.001 | Left superior parietal gyrus |
| 3 | -12 | -12 | 702 | 4.84 | <0.001 | Right substantia nigra |
| -6 | -12 | -18 |  | 3.77 | <0.001 | Left substantia nigra |
| -33 | -24 | 69 | 459 | 4.59 | <0.001 | Left precentral gyrus |
| -24 | -21 | 75 |  | 3.72 | <0.001 | Left precentral gyrus |
| 3 | 9 | -6 | 351 | 4.52 | <0.001 | Right olfactory cortex |
| -21 | -39 | 6 | 513 | 4.18 | <0.001 | Left hippocampus |
| -15 | -30 | -27 | 405 | 4.17 | <0.001 | Left cerebellum |
| 6 | -48 | 72 | 459 | 4.16 | <0.001 | Right precuneus |
| 0 | -48 | 63 |  | 4.00 | <0.001 | Right precuneus |
| 15 | -36 | 48 | 567 | 4.10 | <0.001 | Right paracentral lobule |
| 12 | -42 | 63 |  | 3.55 | <0.001 | Right precuneus |
| 18 | -30 | -27 | 297 | 3.93 | <0.001 | Right cerebellum |
| -18 | -24 | -12 | 675 | 3.59 | <0.001 | Left hippocampus |
| -9 | -33 | -6 |  | 3.57 | <0.001 | Left lingual gyrus |
| -27 | -21 | -15 |  | 3.53 | <0.001 | Left hippocampus |

*Note.* Peak-level activations from the t-contrast Pre > Post of the paired t-test in the PRG1 group, PRG1 = first-time pregnancy group (*n* = 40), Pre = before conception, Post = early postpartum (approx. 3 months).

**Table S9**

*Peak-level activations for the paired t-test Pre vs. Post of mentalizing adult in the PRG2 group*

| MNI coordinates | | | Cluster size (mm^3^) |  |  |  |
| --- | --- | --- | --- | --- | --- | --- |
| x | **y** | **z** |  | **T-value** | ***p*** | **Brain region** |
| 27 | 33 | 45 | 432 | 4.43 | <0.001 | Right superior frontal gyrus |
| 30 | 21 | 48 |  | 3.97 | <0.001 | Right superior frontal gyrus |
| 33 | 33 | 39 |  | 3.36 | <0.001 | Right middle frontal gyrus |
| 18 | -27 | -9 | 270 | 4.32 | <0.001 | Right lingual gyrus |

*Note.* Peak-level activations from the t-contrast Pre < Post of the paired t-test in the PRG2 group, PRG2 = second-time pregnancy group (*n* = 30), Pre = before conception, Post = early postpartum (approx. 3 months).

**Table S10**

*Peak-level activations for the paired t-test Pre vs. Post +1y of mentalizing adult in the PRG1 group*

| MNI coordinates | | | Cluster size (mm^3^) |  |  |  |
| --- | --- | --- | --- | --- | --- | --- |
| x | **y** | **z** |  | **T-value** | ***p*** | **Brain region** |
| -27 | -27 | 66 | 675 | 5.28 | <0.001 | Left precentral gyrus |
| -39 | -24 | 63 |  | 4.59 | <0.001 | Left precentral gyrus |
| -24 | -3 | -27 | 810 | 5.08 | <0.001 | Left amygdala |
| -12 | -6 | -24 |  | 4.02 | <0.001 | Left parahippocampal gyrus |
| -12 | -15 | -21 |  | 3.47 | <0.001 | Left parahippocampal gyrus |
| -9 | -72 | 36 | 1107 | 4.83 | <0.001 | Left precuneus |
| -9 | -78 | 48 |  | 4.73 | <0.001 | Left precuneus |
| -15 | -66 | 27 |  | 3.64 | <0.001 | Left superior occipital gyrus |
| 15 | -30 | -24 | 702 | 4.69 | <0.001 | Right cerebellum |
| 12 | -45 | -15 |  | 3.89 | <0.001 | Right cerebellum |
| 9 | -48 | -39 | 405 | 4.68 | <0.001 | Right cerebellum |
| 3 | -42 | -39 |  | 4.37 | <0.001 | Vermis |
| -6 | -48 | -36 |  | 3.71 | <0.001 | Left cerebellum |
| -3 | -54 | -9 | 648 | 4.57 | <0.001 | Left cerebellum |
| -15 | 9 | -24 | 270 | 4.28 | <0.001 | Left posterior orbital gyrus |
| -24 | 9 | -18 |  | 3.89 | <0.001 | Left insula |

*Note.* Peak-level activations from the t-contrast Pre > Post +1y of the paired t-test in the PRG1 group, PRG1 = first-time pregnancy group (*n* = 40), Pre = before conception, Post +1y = late postpartum (approx. 1 year).

**Table S11**

*Peak-level activations for the paired t-test Pre vs. Post +1y of mentalizing adult in the PRG2 group*

| MNI coordinates | | | Cluster size (mm^3^) |  |  |  |
| --- | --- | --- | --- | --- | --- | --- |
| x | **y** | **z** |  | **T-value** | ***p*** | **Brain region** |
| 15 | -33 | -6 | 648 | 7.11 | <0.001 | Right lingual gyrus |
| 24 | -27 | -9 |  | 4.91 | <0.001 | Right hippocampus |
| -36 | -9 | 9 | 1080 | 5.71 | <0.001 | Left insula |

*Note.* Peak-level activations from the t-contrast Pre < Post + 1y of the paired t-test in the PRG2 group, PRG2 = second-time pregnancy group (*n* = 30), Pre = before conception, Post = late postpartum (approx. 1 year).

**Table S12**

*Peak-level activations for the paired t-test Post vs. Post +1y of mentalizing adult in the PRG2 group*

| MNI coordinates | | | Cluster size (mm^3^) |  |  |  |
| --- | --- | --- | --- | --- | --- | --- |
| x | **y** | **z** |  | **T-value** | ***p*** | **Brain region** |
| -39 | -9 | 3 | 270 | 4.87 | <0.001 | Left insula |

*Note.* Peak-level activations from the t-contrast Post < Post + 1y of the paired t-test in the PRG2 group, PRG2 = second-time pregnancy group (*n* = 30), Post = early postpartum (approx. 3 months), Post = late postpartum (approx. 1 year).

**Table S13**

*Within-group comparisons of the accuracy in the two mentalizing conditions*

|  | CTR group | | PRG1 group | | | | | | PRG2 group | | | | | |
| --- | --- | --- | --- | --- | --- | --- | --- | --- | --- | --- | --- | --- | --- | --- |
|  | **Pre vs. Post** | | **Pre vs. Post** | | **Post vs. Post+1y** | | **Pre vs. Post+1y** | | **Pre vs. Post** | | **Post vs. Post+1y** | | **Pre vs. Post+1y** | |
|  | t(39) / V | *p* | V | *p* | V | *p* | V | *p* | t(29) | *p* | t(13) | *p* | t(13) | *p* |
| mentalizing adult | -2.16 | 0.037* | 260 | 0.72 | 140 | 0.98 | 99 | 0.24 | 0.62 | 0.54 | 0.37 | 0.72 | 0.86 | 0.41 |
| mentalizing child | 357 | 0.17 | 242 | 0.49 | 104 | 0.47 | 100 | 0.15 | -0.94 | 0.36 | -0.58 | 0.57 | -1.22 | 0.24 |

*Note.* Results of the paired t-tests per group of the mentalizing performance accuracy. V is the result of a Wilcoxon signed rank exact test and t is showing the result of a paired t-test. PRG1 = first-time pregnancy group (*n* = 40), PRG2 = second-time pregnancy group (*n* = 30), CTR = nulliparous control group (*n* = 40), Pre = before conception, Post = early postpartum (approx. 3 months), Post + 1y = late postpartum (approx. 1 year). * *p* < 0.05.

**Table S14**

*Within-group comparisons of the accuracy between the two conditions mentalizing adult and mentalizing child*

|  | **Pre** | | **Post** | | **Post+1y** | |
| --- | --- | --- | --- | --- | --- | --- |
|  | V / t(29) | *p* | t(39 / 29) / V | *p* | t(28 / 13) | *p* |
| CTR group | 628 | < 0.001* | 7.56 | < 0.001* | - | - |
| PRG1 group | 642 | < 0.001* | 677 | < 0.001* | 4.73 | < 0.001* |
| PRG2 group | 5.19 | < 0.001* | 4.21 | < 0.001* | 1.23 | 0.24 |

*Note.* Results of the paired t-tests of the mentalizing performance accuracy per group and time point between the two conditions. V is the result of a Wilcoxon signed rank exact test and t is showing the result of a paired t-test. PRG1 = first-time pregnancy group (*n* = 40), PRG2 = second-time pregnancy group (*n* = 30), CTR = nulliparous control group (*n* = 40), Pre = before conception, Post = early postpartum (approx. 3 months), Post + 1y = late postpartum (approx. 1 year). * *p* < 0.001.

**Table S15**

*Between-group comparisons of the mentalizing child and mentalizing adult accuracy*

|  | **Pre** | | **Post** | | **Post+1y** | |
| --- | --- | --- | --- | --- | --- | --- |
|  | V / t (df) | *p* | V / t (df) | *p* | V | *p* |
| **mentalizing adult** |  |  |  |  |  |  |
| CTR vs. PRG1 | -0.84 (77.1) | 0.40 | 857 | 0.58 | - | - |
| CTR vs. PRG2 | -1.06 (60.5) | 0.29 | 1.27 (62.4) | 0.21 | - | - |
| PRG1 vs. PRG2 | -0.25 (64.1) | 0.80 | 657 | 0.50 | 492 | 0.39 |
| **mentalizing child** |  |  |  |  |  |  |
| CTR vs. PRG1 | 868 | 0.51 | 709 | 0.38 | - | - |
| CTR vs. PRG2 | 611 | 0.90 | -1.71 (66.4) | 0.09 | - | - |
| PRG1 vs. PRG2 | 567 | 0.68 | 493 | 0.20 | 196 | 0.85 |

*Note.* Results of the two sample t-tests of the mentalizing performance accuracy per time point and condition between the groups. V is the result of a Wilcoxon signed rank exact test and t is showing the result of a paired t-test. PRG1 = first-time pregnancy group (*n* = 40), PRG2 = second-time pregnancy group (*n* = 30), CTR = nulliparous control group (*n* = 40), Pre = before conception, Post = early postpartum (approx. 3 months), Post + 1y = late postpartum (approx. 1 year).

Supplementary Figures


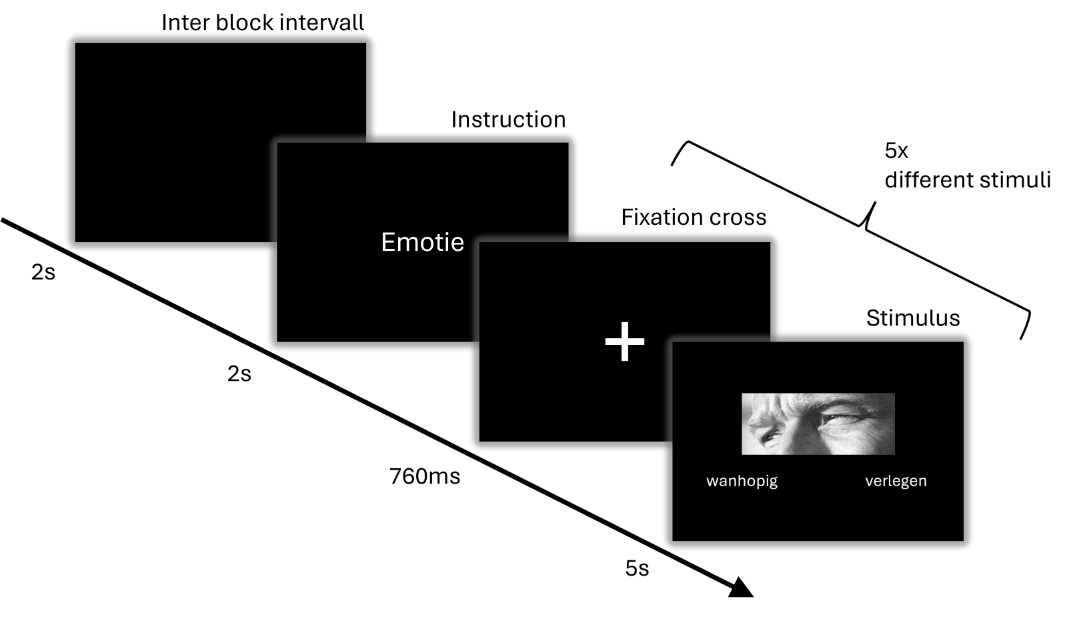


**Figure S1. Reading the Mind in the Eyes paradigm.** Visualization of the task used during the fMRI. There were alternating blocks of either the emotion (mentalizing) or the gender (control) condition. Each block consisted of five different stimuli. The set up is exactly the same for the Reading the Mind in the Child’s Eyes task, just the pictures are showing childrens eyes.


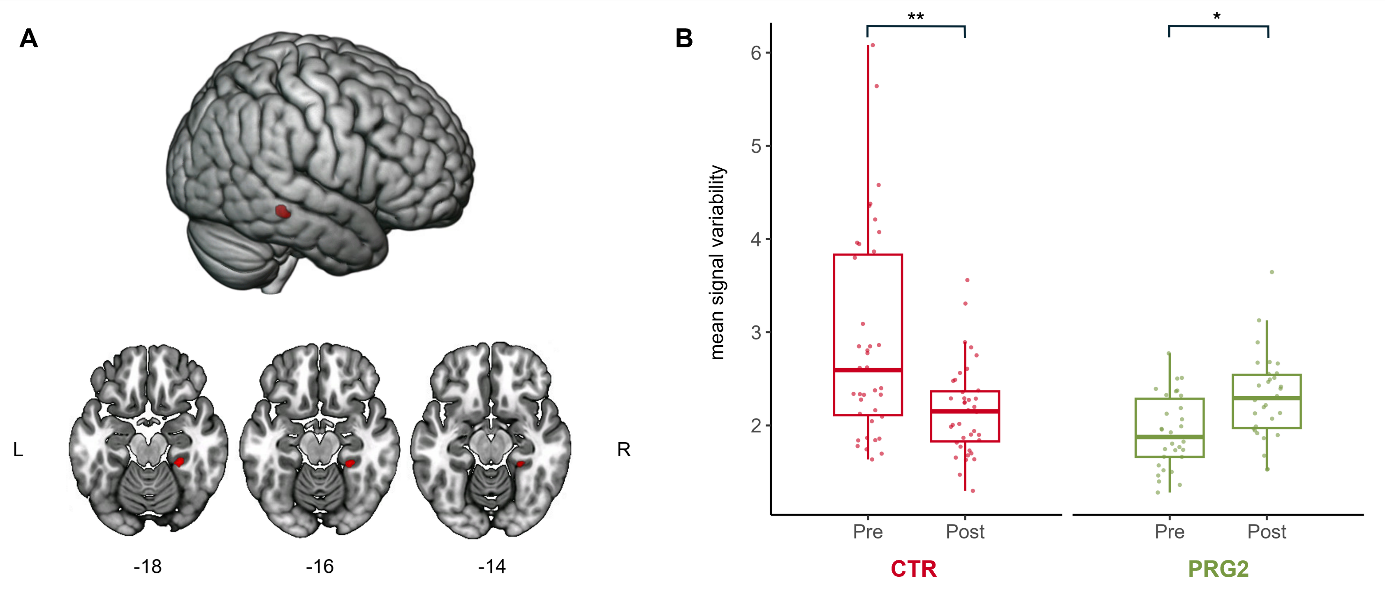


**Figure S2. Neural variability changes over time in the group-by-time interaction cluster in the right fusiform gyrus during the mentalizing adult condition.** Visualization of the cluster location from the interaction model for CTR vs. PRG2 and the group distribution per time point. CTR = control group (*n* = 40), PRG2 = second-time pregnancy group (*n* = 30), Pre = before conception, Post = early postpartum. (A) shows the location of the interaction cluster. (B) shows the mean neural variability per time point for each group. Boxplots show the median and interquartile range (IQR); whiskers extend to 1.5x IQR. Individual data points (jittered) are overlaid for visualization and include all values. ** *p* < 0.001, * *p* < 0.05.
